# Development of Hospital Perception Scale for Healthy Children (HPSHC) and Investigation of Its Psychometric Properties

**DOI:** 10.3390/children10101706

**Published:** 2023-10-20

**Authors:** Behice Ekici

**Affiliations:** School of Nursing, Maltepe University, Istanbul 34857, Türkiye; behiceekici@maltepe.edu.tr

**Keywords:** perception, child, hospital, perception of hospital, scale development

## Abstract

(1) Aim: The aim of the study was to develop and analyze the psychometric properties of a hospital perception scale for healthy children aged 8 to 10 years. (2) Methods: A methodological design was employed. The scale’s validity was investigated using the approaches of content validity, face validity, item analysis, and construct validity. The scale’s reliability was evaluated utilizing the approaches of internal consistency reliability, measurer reliability, and measurement invariance. (3) Results: In total, 330 children took part in this study. The scale is composed of six factors. Factor loads range from 0.42 to 0.79. The item–total score correlation coefficients were 0.42 and 0.79, respectively, while the Cronbach alpha reliability coefficient was 0.87. (4) Conclusions: The HPSHC is a valid and reliable tool. It can be used to determine how healthy or sick children in their middle childhood are perceived when going to the hospital and being hospitalized.

## 1. Introduction

Children require hospital visits for diagnosis, treatment, and short- or long-term hospitalizations. Children’s perceptions of hospitals are shaped by their developmental level, indirect knowledge learned about hospitals in the past, and their own experiences [1,2]. If a child has been hospitalized previously and this hospitalization was associated with an invasive procedure, suffering, discomfort, fear, or a great deal of crying, they will probably have the same fears in another encounter. Some children even fear becoming disabled or dying [3,4,5,6,7]. For example, several studies have demonstrated that children commonly exhibit elevated levels of anxiety during their hospitalization [4,8,9]. Research has indicated that adverse childhood experiences are associated with detrimental effects on both physical and psychological well-being [10].

Children’s perceptions toward hospitals differ depending on their developmental level and needs. For example, children aged 7 to 10 have distinct ways of thinking since they begin to develop logical thinking skills at this age. They begin to distinguish themselves from their surroundings. The need for autonomy and control over one’s own body and life grows, as does the importance of social surroundings. The development of adult thought begins around the age of eleven [11,12,13].

In this study, it was deemed important to examine the perception of hospitals of 8- to 10-year-olds with substantial hospital experience whose thinking styles and demands have evolved. During a child’s illness or hospitalization, the focus is typically on the treatment and care of the disease, but the child’s views of the process are frequently overlooked. Other than the child medical fear scale (CMFS), developed by Broome and Hellier in 1987 [14], we were unable to identify a scale that measures the perception of hospitals of children aged 7 to 10 or other ages in the literature. This Turkish version of the scale was first published in 1993. Although useful in the past, the CMFS scale’s narrow focus on anxiety about medical procedures and its somewhat dated publication date indicate that a more modern instrument is required for accurate assessment. Due to a lack of a validated instruments in the existing literature, we set out to create a measurement tool that would allow us to gauge how hospitals are perceived by healthy children. The perception of a hospital is influenced by a child’s developmental stage and their indirect knowledge or personal experiences with hospitals in the past [2]. The aim of this study was to evaluate the general perception of hospitals of healthy children based on their past experiences.

Based on this primary aim, the study’s objective was to develop an instrument that can measure the perception of hospitals of healthy children aged between 8 and 10 years, who have previously been hospitalized for examination, follow-up, or outpatient treatment, or who have experienced short-term hospitalization (e.g., 1–3 days) due to acute illness. It was also intended to investigate and validate the psychometric features of the proposed scale. In this study, the hypothesis “HPSHC is a valid and reliable instrument for measuring healthy children’s hospital perceptions” was tested.

## 2. Material and Methods

### 2.1. Study Design and Population

#### 2.1.1. Study Design

**Figure 1:**
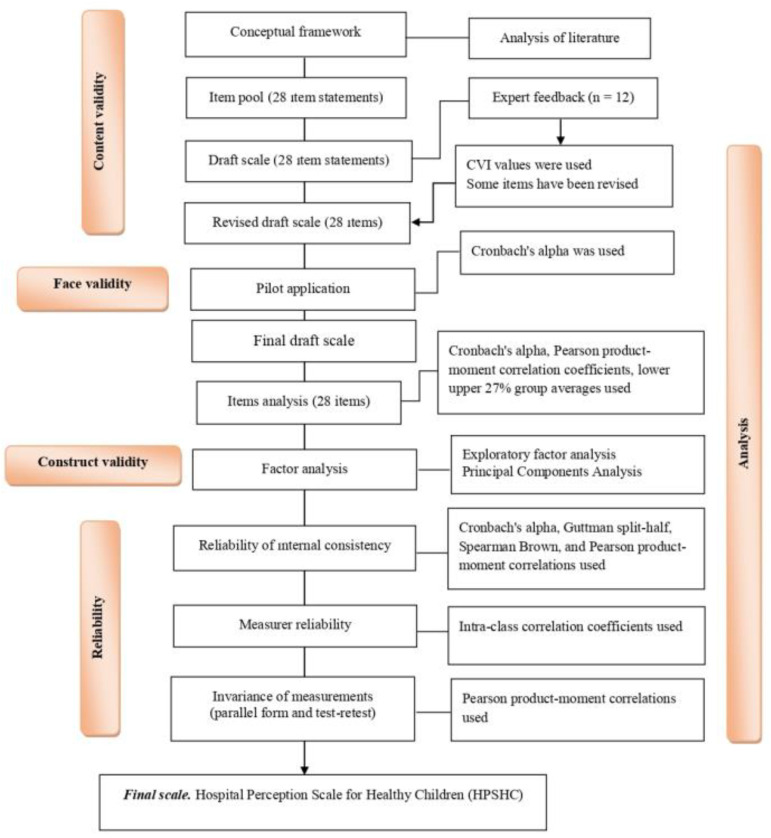
The HPSHC development and evaluation process.

The HPSHC was created in this study with a methodical approach, and its psychometric qualities were investigated (Figure 1).

#### 2.1.2. Settings and Population 

The primary objective of this study was to develop a comprehensive scale for assessing perceptions of general hospitals. To achieve this goal, the survey was carried out among a sample of healthy children within the school setting. The study was conducted between November and December 2021 in an randomly chosen district of Istanbul, Turkey, and in five randomly chosen public primary schools within this district’s center. The lottery method was used to randomly choose the district and schools where the study would be conducted. The objective was to minimize the impact on the overall perception of unwell children in hospitals as they undergo treatment and cope with the consequences of their illness. The present study was carried out in public schools (free education) so that the potential impact of variations in children’s social and cultural backgrounds on their perceptions of hospitals was mitigated. Children attending the schools were from an urban population. Based on their self-disclosed remarks, the households possess a moderate level of income. The schools were primary education institutes. Both female and male students are enrolled in the second, third, and fourth grades of primary education. The children’s ages range from 8 to 10 years old.

A sample size of 300 was taken, based on the opinion of Henson and Roberts [15]. The study enrolled 460 school-aged children that attended school on the date of data collection. Briefly, 330 of these children met the inclusion criteria and were thereafter included in the study. 

The HPSHC was administered to the same participants (330 children) a second time two weeks later to calculate reliability coefficients using the test–retest procedure. The majority of the children attended school remotely on the date of the second measurement of the scale (7 December 2021), as schools returned to hybrid education because of the COVID-19 pandemic. As a result, 100 of the 330 children who took part in the initial measurement (100 children) took part in the retest. Therefore, test–retest analysis was computed using data from 100 students. Briefly, 39 of these children were eight years old, 38 were nine years old, and 23 were ten years old. There were 49 females and 51 males.

#### 2.1.3. Inclusion and Exclusion Criteria

During the establishment of the study’s inclusion criteria, careful consideration was given to the requirement that participants be 7 years of age or older, as this age represents the onset of logical thinking skills required for a proper understanding and interpretation of the scale items. In addition, it was essential that the children selected for the study be in excellent health and without chronic illnesses at the time they completed the scale form. The inclusion criteria for the study were as follows: (1) children between the ages of 8 and 10, (2) previous hospitalization for evaluation, observation, or outpatient treatment, (3) short-term (1–3 days) hospitalization for acute illness, (4) attendance at school on the day of data collection, (5) an ability to read and respond to the items on both scales, (6) completion of the data collection forms, (7) parental consent to participate in the study, and (8) an absence of chronic disease. Children of parents who did not consent to participation in the study (n = 51), children with a chronic disease (n = 14), and children who did not complete the data collecting forms (n = 65) were excluded from the study. Children with a chronic disease were excluded from the study because of permanent or long-term physical disability, and it was believed that the perception of hospitals of these children would be significantly different from that of healthy children. Due to hospital visits or hospitalization, these children must frequently be apart from their family, friends, and familiar environments, which can alter their self-perception and lead to low self-esteem. Furthermore, some children may believe they will not get better, will become disabled, or even die. In addition, these children frequently experience pain and anxiety as a result of the monitoring, treatment, and care of their chronic diseases [1,4,8,9,10]. 

### 2.2. Data Collection

#### 2.2.1. Procedure 

The following steps were taken for data collection. Initially, the researcher conducted an interview with school administrators via telephone. During this call, information was provided regarding the research and relevant permits, and the dates for the first and second school visits for data collection were set (1st visit: 23 November 2021; 2nd visit: 7 December 2021). The process of study and data gathering was described to the classroom teachers of the students. The number of students in each class was noted. On the first day, teachers provided each child with one copy of the data collecting instruments (the informed consent form, CIF, HPSHC, and CMFS). The children were asked to take the data collection instruments to their parents, and those whose parents consented completed and returned the questionnaires the next day. The children were asked to respond to the questions on the data collection form by recalling previous hospital visits.

In addition, the teachers sent brief information to a messaging group composed of the kids’ parents. In the communication, parents were asked to review the submitted forms, have their child complete the papers if they gave permission, and return the forms the following day with the student. The following day, data collection instruments were obtained from the teachers of the students. Two weeks later, the HPSHC was administered for the second time to all children who participated in the first application. In this application, the same procedures as those in the first measurement were applied. 

#### 2.2.2. Instruments

The child information form, HPSHC, and CMFS were used to collect data.

**Child Information Form (CIF)**

The researcher developed this form to gather information on the health status and socio-demographic features of the children. On this form, five questions were used to record the participants’ age, gender, presence of any disease, grade of attendance, and the response time of the participant to each scale.

**Hospital Perception Scale for Healthy Children (HPSHC)**

This scale was designed by the researcher to assess the perception of hospitals of children aged 8 to 10. At the beginning of the scale is a description of how the form should be filled out. Following the explanation are the item statements constructed in accordance with the conceptual structure. According to the child’s level of agreement with the item statement, the options for each item were constructed using a 3-point Likert scale. “*I never think like this*” is worth 0 points, “*I sometimes think like this*” is worth 1 point, and “*I always think like this*” is worth 2 points.

**Child medical fear scale (CMFS)**

Broome and Hellier originally designed the scale for use with children aged 7 to 14 years [14], then Alak validated the scale in the Turkish language [16]. There are 29 items in total on the scale. The 4-factor structure of the scale includes procedural (9 items), environmental (7 items), personal (4 items), and interpersonal (9 items) sections. The items included in the scale express statements of fear regarding certain aspects related to the healthcare environment, which the child must answer according to a Likert-type scale with three points: not at all (0 points), a little (1 point), and a lot (2 points). The rating system has a score range and cutoffs. The scale has a minimum of 29 points and a maximum of 87 points. A child’s overall score on the scale corresponds to the following: 0–29 points = little fear; 29–58 points = some fear; and 58–87 points = extreme fear. Studies have found the Cronbach’s α coefficient of the CMFS to be between 0.78 and 0.93 [14,16,17,18,19]. 

### 2.3. Scale Development Process

For the construction of the scale and evaluation of its psychometric features, theoretical and methodological investigations of validity and reliability were conducted.

#### 2.3.1. Content Validity 

Content validation is carried out in two stages. In the first stage, the measuring area is determined, and the conceptual framework and item pool are created in accordance with this determination [20,21,22,23]. 

In the second stage, to obtain expert opinion, a 3- or 4-point Likert-type scale draft is created. Then, a group of at least three specialists with knowledge and experience in the measurement field is assembled. Experts are requested to evaluate whether or not each item on the draft scale is relevant to the measure. Methods are employed to convert expert opinions into quantitative data by calculating the content validity rate (CVR) and content validity index (CVI) of the items. According to the index breakpoints, a decision is made as to whether or not the items will continue in the proposed scale form [20,23,24,25,26,27]. The technique of Polit et al. (2007) is frequently used in transforming expert opinions into quantitative data [27]. In this technique, the item content validity index (I-CVI), mean content validity index (Ave-CVI) and universal agreement content validity index (UA-CVI) values of each item are calculated. The I-CVI value is calculated by dividing the sum of the number of experts who awarded each item 3 or 4 points by the total number of experts. The Ave-CVI value is calculated by adding the I-CVI values of every item and dividing it by the total number of items. The UA-CVI value, on the other hand, is determined by dividing the total number of items awarded 3 or 4 points by all experts by the total number of items [27].

#### 2.3.2. Face Validity

For face validity, the participants’ difficulties in reading and comprehending the item statements on the scale form and in selecting the appropriate response option is assessed in a pilot application [21,23,28]. 

#### 2.3.3. Item Analysis

Before item analysis, the mean, standard deviation, minimum and maximum scores are determined for each item. The significance (*p* < 0.05) of the difference in item means is determined using Hotelling’s T^2^ analysis. In the data distribution, it is suggested that the skewness and kurtosis values fall within the range of ±1.5 [28,29]. The item analysis of the scale is evaluated using the mean difference between the lower and upper 27% of the groups (*t*-test for independent samples) [21,28,30]. 

#### 2.3.4. Construct Validity 

Exploratory factor analysis (EFA) is utilized in the construct validity analysis of the scale to determine the constructs that the scale assesses [22,23,31,32,33,34]. Before EFA, the conformity of the data to EFA is evaluated with the Kaiser–Meyer–Olkin (KMO) test and Bartlett’s sphericity χ^2^ test. In this analysis, the KMO value should be >0.60 and in Bartlett’s test of sphericity, the *p* value should be <0.05 [15,22,29,31]. To determine the number of factors, the eigenvalue is checked and the factors with an eigenvalue of ≥1.0 are considered. Items with a factor loading of ˂0.30 and items with a factor loading of ˂0.10 are excluded from the scale [15,21,22,33]. 

#### 2.3.5. Reliability Analyses 

Internal consistency reliability, measurer reliability, and measurement invariance are assessed during reliability analyses.

**Reliability of Internal Consistency**

*Calculation of Internal Consistency Reliability Coefficients.* Internal consistency reliability is assessed by calculating Cronbach’s alpha, the Guttman split-half, and Spearman–Brown reliability coefficients. It is determined based on the score acquired from the entire scale, its factors, and the two halves produced by dividing the scale into two halves of equal size [23,28,29].

*Calculation of Item–Total Score Correlation Coefficients.* In item–total score correlation, the Pearson product–moment correlation coefficient (PPMCC) is calculated [21,28,29,30].

**Measurer Reliability**

*Calculation of Intraclass Correlation Coefficients.* Using the intraclass correlation coefficient (ICC), the consistency between repeated measurements of the same continuous and normally distributed variable with the same participants is evaluated. Using the analysis of variance (ANOVA) test and the two-way mixed effect model, the ICC coefficient is computed. The coefficient of the ICC is derived based on the average intraclass correlation scores of many observers [23,28,29,35].

**Invariance of Measurements**

In the investigation of the relationship between two variables, PPMCCs are obtained when the measurements are invariant. Calculated coefficients are considered reliability coefficients [23,28,29,30]. 

*Calculation of Reliability Coefficients with the Parallel Form Method.* The newly designed and previously developed scales, which measure the same condition, are applied simultaneously and sequentially. The mean, standard deviation, and variance of both scales must be the same. The correlation coefficients between the total scores of both scales and the total scores of the items collected under the factors are calculated [21,28,30].

*Calculation of Reliability Coefficients with the Test–Retest Method.* The interval approach is used to deliver the scale to the same participants twice, and the invariance of the two measurement scores is verified against time. The correlation coefficients between the total scores of the scales obtained in both measurements and the total scores of the items collected under the factors are calculated [21,23,28].

### 2.4. Evaluation of the Scale 

IBM Statistical Package for Social Science (SPSS) Statistics for Windows version 21.0 was used to analyze the data. This study employed SPSS for conducting EFA to ensure the following: (1) that the responses were evenly distributed, (2) the participants were selected through random sampling and were representative of the entire population, (3) the sample size was adequate, (4) the responses were measurable, and (5) there existed a linear relationship between the responses and the data followed a normal distribution [29,36]. It was ascertained through the normality analysis that the data followed a normal distribution.

Minimum (min), maximum (max), mean, standard deviation (SD), number, and percentage are reported for the data. Each item’s mean, SD, skewness, and kurtosis values were used to examine the distribution of the data. For the scale’s content validity, the CVI values of the experts’ opinions were determined. Item analysis was performed by comparing the means between the upper and lower 27% of the groups (*t*-test for independent samples). Construct validity was evaluated using factor analysis. For the investigation of the scale’s internal consistency reliability, Cronbach’s alpha, the Guttman split-half, Spearman–Brown reliability coefficients, and item total score correlation coefficients were determined. ANOVA was used to calculate ICC coefficients for measurer reliability. For the invariance of the measurements, reliability coefficients (PPMCCs) were generated and evaluated using the parallel form and test–retest method.

### 2.5. Ethical Considerations

The research was approved by Maltepe University Ethics Committee (approval no: 2020/02-28; approval date: 4 October 2020). Research permission was obtained from the institution of the schools where the research data were collected (permission no: E-59090411-20-36602234). Written informed consent was obtained from the parents who voluntarily allowed their children to participate in the study. The informed consent form was used to inform the parents of the participating children of the purpose of the study, the voluntary nature of participation, the ability to withdraw from the study at any time, the fact that participants would complete the scales twice, with a two-week interval between each, and that data would be used exclusively for this study.

## 3. Results

### 3.1. General Characteristics of the Participants

Three hundred and thirty children participated in this study. Of the participants, 110 (33.3%) were 8 years old, 169 (51.2%) were 9 years old, and 51 (15.5%) were 10 years old. The mean age of the children was 8.82 ± 0.67 years old. There were 186 (56.4%) females and 144 (43.6%) males. All participants attended elementary school, of which 16 (4.8%) attended second year, 143 (43.3%) attended third year, and 171 (51.8%) attended fourth year. The children read the items on each scale and then selected the option that best reflected them. For each scale, the response time of the youngsters was similar (average: 6.0 ± 1.2; range: 4–8 min).

### 3.2. Content Validity Studies and Analyses of the Draft Scale

#### 3.2.1. Creating the Item Pool of the Draft Scale

The item pool was developed based on a thorough examination of the literature and the researcher’s 36 years of clinical and academic experience. There were 28 item statements in the item pool that comprised concepts related to the perception of hospitals of children.

#### 3.2.2. Creation of the Draft Scale

A directive was added to the top of the draft scale. In the directive, experts were asked to rate each item according to how they thought the item would measure a child’s perception of a hospital (by giving points) and to also write down their suggestions, if any. Just below the directive was the item pool. Experts rated each item on the scale using a four-point Likert scale, in accordance with the technique of Davis (1992) [25]. For this purpose, four columns were added opposite each of the 28 items, for scoring. The Likert scale was as follows: 1 point = not relevant, 2 points = somewhat relevant, 3 points = highly relevant, and 4 points = extremely relevant. A line was added under each item for experts to write their suggestions.

#### 3.2.3. Creation of the Expert Group

A panel of twelve experts focused on the developmental characteristics of children and the relationship between children and disease, and the hospitalization of children was formed. The expert panel consisted of three physicians, three clinical nurses, four academic pediatric nurses, and two academics working in the field of child development and education. Pediatricians, clinicians, and academic nurses often possess a minimum of 10 years of professional experience within pediatric healthcare organizations. Academics specializing in the domain of child development and education have frequently engaged in educational institutions providing early childhood, primary, and secondary education for at least two decades.

#### 3.2.4. Obtaining and Evaluating Expert Opinions

The draft scale was e-mailed to the experts. After the experts reviewed the form, they e-mailed it back to the researcher. The technique of Polit et al. (2007) was applied in the evaluation and interpretation of expert opinions [27]. Three experts suggested minor adjustments to the phrasing of the first 19 items. After adjusting the items based on feedback, the form was re-sent to the experts a second time for their feedback. Following the initial evaluation, the phrasing of the first 19 items was modified based on the advice of three experts. For example, Item 1 was modified from “I think I shall be separated from my family in hospital” to “When I go to the hospital, I think I will be separated from my family”. The adjusted draft scale was delivered again to the same experts for a second evaluation (10 experts). The alterations made to the item expressions were deemed reasonable by all experts, who did not alter the initial item scores. Each item’s I-CVI values were found to be between 0.83 and 1.00 based on the evaluation findings of the experts. All items on the draft scale had an Ave-CVI value of 0.96 and a UA-CVI value of 0.71. During the first and second examinations, none of the experts awarded the items a score of 1 point.

### 3.3. Face Validity Analyses of the Draft Scale

A pilot study was conducted with the participation of 24 children (7 children were 8 years old, 9 were 9 years old, and 8 were 10 years old) who met the inclusion criteria. The children had no difficulty reading, comprehending, or selecting the item statements in the scale.

### 3.4. Item Analyses of the Draft Scale

The average score on the 28-item scale form was 46.92 ± 9.88 (range 28–77). Hotelling’s T^2^ analysis demonstrated a statistically significant difference between the means of the items (*p* < 0.0001). Data were considered to be normally distributed as both the skewness (0.330) and kurtosis (−0.303) values fell within the range of ±1.5. Positive and statistically significant differences existed between the item scores and scale item total score averages for the lower (31.89 ± 2.95) and upper (54.53 ± 4.91) 27% of the scale (t = 7.099, *df* = 176; *p* = 0.0001).

### 3.5. Construct Validity Analyses of the Draft Scale

#### Exploratory Factor Analysis

The KMO value for the 28-item EFA was 0.864 (>0.60), and the p value for the Barlett test of sphericity was <0.0001 (χ^2^ = 2418.574; *df* = 378). In terms of construct validity, EFA was conducted four times. Item 6 (I believe I will be separated from my toys when I go to the hospital) and item 8 (I believe I will be punished when I go to the hospital) were removed after the first and second EFA based on 28 and 27 items, respectively, because they were not loaded on any of the variables. Item 28 (I believe I cannot sleep comfortably in the hospital) was removed after the third EFA based on 26 items since it loaded from two factors and the difference between the load values was <0.10. In the fourth EFA based on 25 items, the KMO value was found to be 0.867 and the p value for the Barlett test of sphericity (χ^2^ = 2136.154; df = 300) was found to be < 0.001. The breakpoints/eigenvalues of the variance explained by each factor in the EFA (variance) analysis are ≥1.0. Eigenvalues were calculated to be 1st factor = 6.09, 2nd factor = 2.04, 3rd factor = 1.81, 4th factor = 1.29, 5th factor = 1.11, and 6th factor = 1.02 based on the scree plot. The findings of the EFA (variance) analysis are shown in Table 1. The first factor made up 24.36% of the variance (the highest variance). The second factor accounted for 8.16%, the third element for 7.26%, the fourth factor for 5.19%, the fifth factor for 4.47%, and the sixth factor for 4.10% of the variance. The analysis of the six-factor structure revealed that none of the items exhibited a significant influence from multiple factors, as indicated by the factor loadings ranging from 0.42 to 0.79 (Table 1). The communality values of the components in the six-factor framework are less than 1 (range 0.37–0.65). The six-factor structure accounts for 53.57% of the total variance. 

### 3.6. Reliability Analyses of the Scale

Prior to the reliability study, Hotelling’s T^2^ analysis of 25 items revealed a statistically significant difference between the item means (*p* < 0.0001).

#### 3.6.1. Internal Consistency Reliability Analysis

*Calculation of Internal Consistency Coefficients.* The scale’s overall Cronbach’s alpha coefficient was 0.87 (range = 0.58 to 0.76) (Table 1). The Guttman split-half approach yielded Cronbach’s alpha coefficients of 0.80 for the first 13 items of the scale and those of 0.77 for the final 12 items. The correlation value between the two scale variants containing the first 13 and last 12 items is 0.69. The scale’s Guttman split-half reliability coefficient is 0.81. The coefficients for the equal and unequal Spearman–Brown relationships are 0.82 (Table 2).

*Calculation of Item–Total Score Correlation Coefficients.* The correlation between the item–total scores of the scale was positively significant (*p* < 0.05). Correlation coefficients were between 0.32 and 0.57 (Table 1).

#### 3.6.2. Measurer Reliability Analyses

*Calculation of Intraclass Correlation Coefficients.* For the two measures, the scale total scores and total factor scores differed significantly from the average measure intraclass correlation (*p* < 0.0001). The ICC value for the correlation between the total scores of the scale was 0.79, while the ICC values for the correlation between the total scores of the factors ranged from 0.53 to 0.83 (Table 3).

#### 3.6.3. Analysis of Invariance of Measurements

*Parallel Form Reliability Analysis*. Prior to analysis, the equality criterion for the mean and standard deviation values of the 28-item HPSHC (46.92 ± 9.88) and 29-item CMFS scales (45.24 ± 10.16) was examined. The variance values of the scales (HPSHC = 97.733; CMFS = 103.316) were similar. Positively significant correlation coefficients (*p* < 0.0001) were found between the scale total scores of the 25-item HPSHC and the 29-item CMFS and the total scores of the respective components (Table 4).

*Test–Retest Reliability Analysis.* The coefficients of the correlation between the total scale scores obtained from the two measures and the total factor scores were found to be statistically significant in the positive direction. (*p* < 0.0001) (Table 3). 

### 3.7. Scoring of the Scale

The 25-item scale has a total score range of 0 (min) to 50 points (max). The total score is interpreted as follows: 0–24 = “*less negative hospital impression*”; 25–50 = “*a great deal of negative hospital perception*”. A higher score implies that the youngster has a more negative opinion of hospitals.

## 4. Discussion

Scale development studies are widely utilized in pediatric nursing to assess situations that cannot be directly measured or witnessed. However, no scale has been developed to measure how children interpret hospitalization, which may be the first crisis they face. To this end, only one scale that measures children’s fear of medical procedures is available in the literature. The HPSHC was designed with the purpose of assessing the perception of hospitals among healthy children between the ages of 7 and 10. However, it was necessary to exclude 7-year-old children from the sample due to their insufficient reading and questionnaire-answering skills. In this study, a valid and accurate scale was developed to assess how 8- to 10-year-old healthy children evaluate hospitalization, the hospital environment, and hospital procedures. 

In this section, the outcomes of the theoretical, methodological, and statistical analyses conducted during the process of scale development are discussed. The results of the analysis were examined considering the pertinent literature, and it was determined whether or not the scale met the standards for validity and reliability.

### 4.1. Content Validity of the Scale 

In scale development studies, an item pool is first generated. To determine if the item pool is relevant to the measuring area, expert opinions are solicited. Expert views (qualitative data) are translated into quantitative data by computing CVI values, and the index limit values are used to determine if they meet the content validity criteria [20,21,22,23,37]. It is accepted that the I-CVI and Ave-CVI values of items that will stay in the draft scale should be 0.80 and 0.90, respectively. Items not meeting these requirements are removed from the draft scale [24,26,27]. I-CVI index values in this study ranged from 0.83 to 1.00. All items had an average CVI value of 0.96. In this study, the first and second evaluation outcomes for the 28-item draft scale with I-CVI values ≥ 0.80 and Ave. CVI values ≥ 0.90 were compared. All experts agreed that 28 items on the draft scale were associated with children’s perceptions of illness, and no more item statements were included. Since the I-CVI values of each item of the draft scale and the Ave-CVI values of all items exceeded the index’s lower limit value, it was determined that the draft scale satisfied content validity conditions. The 28-item draft was approved as the final form.

### 4.2. Face Validity of the Scale

In the pilot application of this study, healthy children had no problems reading and understanding the item expressions and marking the options in the 28-item scale form.

### 4.3. Construct Validity of the Scale

In factor analysis, the sample size must be sufficient to produce a stable factor structure. In the literature, sample size recommendations state that if the number of items is 40, at least 200 participants or 20 times the number of items is the optimal sample size (the2 0:1 rule) [21,31,32,33,38]. According to Henson and Roberts, a minimum of 300 participants is often sufficient [12]. This study’s sample included 330 youngsters who met the inclusion criteria. In the EFA, 330 healthy children’s data were analyzed. The sample size for the final factor analysis based on 25 items was 13 times the number of items (the number of participants multiplied by the number of items equals 13:1). This ratio indicates that the minimum sample size requirement was reached and that a stable factor structure was achieved.

Prior to factor analysis, the suitability of the data for EFA was tested according to the KMO value (>0.60) and the Bartlett sphericity χ^2^ test result (*p* ˂ 0.05). In factor analysis, related objects are brought together to generate a small number of new structures (factors). According to the factor loads and the limit values, the resulting structure is analyzed and interpreted. The limit values for factor load are as follows: ≥0.71 for excellent, 0.63 for very good, 0.55 for good, 0.45 for acceptable, and 0.32 for poor. In addition, a factor must encompass at least three things [15,21,22,32,33]. In addition, items with factor loadings of <0.30 and <0.10 are eliminated from the scale [15,21,22]. 

The initial analysis of the study was conducted using a dataset consisting of 330 participants. The 28-item scale utilized exhibited two items (namely, items 6 and 8) that displayed factor loadings below the threshold of <0.30 and a further item (item 28) was found to load on two factors. This led to the exclusion of these items during the factor analysis procedure as they failed to satisfy the analysis requirements. The correlation values between the total scores of these items (Item 6 = 0.28 and Item 8 = 0.29) were also below 0.30.

The final EFA and reliability analyses of the scale form were conducted on 25 items. In the factor analysis of the HPSHC based on 25 items, the factor loads of the items other than the seventh item (factor load = 0.42) were determined to be “*good*”, “*very good*”, and “*excellent*”. In accordance with the literature, the six-factor scale comprises at least factors 4, 5, and 6 or more than three items (Table 1).

The common variance values of each item should be less than 1 and the total explained variance in multi-factor structures should be 50% or more [22,31,32,33]. It was established that the common variance values of the HPSHC were <1 (range: 0.37–0.65) and that the overall explained variance was 53.57% of the scale. Since the six-factor structure was consistent with the 25-item factor analysis, it was deemed valid.

### 4.4. Naming the Factors of the Scale

The names of the factors correspond to the meanings stressed by the item expressions gathered under the EFA’s identified factors. It is determined by examining the common theme of the item with the highest factor loading and the other item statements when identifying the factors [22,37,39]. In this study, naming the factors was straightforward because the 25 items grouped under the factors in the EFA represented the same or comparable notions (Table 1). Consequently, the items collected under factor 1 include perceptions that the child will leave his friends, family, and school when he goes to the hospital and be alone. Therefore, the component was named “*separation*”. The items collected under factor 2 include perception statements regarding the interventions made in the hospital for diagnosis, treatment, follow-up and care. Therefore, the factor was named “*invasive and non-invasive interventions*”. The items collected under factor 3 include the child’s anxieties over hospital interventions and his perceptions of the loss of control over his own bodily interventions. Therefore, the element is referred to as “*fear and loss of control*”. The 4th factor was titled “*body image*” since the data collected under this factor included remarks that the child’s body image would worsen, and that they would die if they went to the hospital. The items collected under factor 5 include perceptions that the child cannot achieve his basic needs, such as defecation, play, and sleep, when he is hospitalized. Therefore, the element was referred to as “*physiological needs*”. The impression statements that make up the items collected under factor 6 describe the hospital environment. Therefore, the component is referred to as “*hospital environment*”.

The loadings of the items gathered under the factors, the explained variance of the factors, and the ranking of the factors were consistent with those in the literature on how children in the middle childhood perceive and respond to hospital visits and hospitalization [1,4,40,41,42]. In addition, the multi-factor structure that emerged in the factor analysis explains in all aspects how healthy children between the ages of 8 and 10 perceive going to the hospital and being hospitalized.

### 4.5. Reliability of Scale

Analyses of internal consistency reliability, measurer reliability, and measurement invariance should be conducted during the scale creation procedure. Internal consistency reliability examines the degree to which the components of a scale function consistently with one another. It is desirable that the Cronbach’s alpha coefficient be ≥ 0.80, and that the Guttman split-half and Spearman–Brown coefficients be ≥0.70 [21,28,30]. The general Cronbach’s alpha coefficient (α = 0.87) of the HPSHC revealed high internal consistency. In accordance with the Guttman split-half approach, that the Cronbach’s alpha values of the first 13 items (α = 0.80) and the last 12 items (α = 0.77) of the scale were near and high indicated that the items were organized sequentially. As the Guttman split-half and Spearman–Brown coefficients for the correlation between the first 13 and last 12 items of the scale were >0.80, the entire scale was deemed reliable (Table 2). According to these findings, the internal consistency reliability is good, as the HPSHC items measure with a high degree of consistency.

In internal consistency analyses, item discrimination is interpreted according to the breakpoints of the item–total score correlation coefficients. The correlation coefficient breakpoints are 0.01–0.19 = absent or negligible, 0.20–0.29 = weak, 0.30–0.39 = moderate, 0.40–0.69 = strong, and ≥0.70 = very strong. Items with a correlation coefficient of minus or ˂0.30 are excluded from the scale because of poor discrimination [21,28,30]. Since the HPSHC item–total score correlation coefficients were positive and >0.30, no items were eliminated from the scale (Table 1). The internal consistency of the scale is between “*moderate*” and “*high*”. According to these results, the items’ discrimination is adequate.

In measurer reliability, an ICC value > 0.75 is recommended to ensure invariance between two measurements. ICC breakpoints are interpreted as ˂0.50 = poor, 0.50–0.75 = fair, 0.75–0.90 = good, and >0.90 = excellent [28]. In two applications of the HPSHC, a “*good*” level of concordance between scale total scores (ICC = 0.79) and factor 1 total scores (ICC = 0.83) was found. The total scores of factors 2–6 (ICC values = 0.53 to 0.72) were in “*moderate*” concordance (Table 3).

In the analysis of the invariance of the measurements, the PPMCCs which are calculated based on the repeated measurement data of the parallel forms and the developed scale are interpreted. The correlation coefficient breakpoints are 0.01–0.19 = absent or negligible, 0.20–0.29 = weak, 0.30–0.39 = moderate, 0.40–0.69 = strong, and ≥0.70 = very strong. In this analysis, it is recommended that the correlation coefficients be positively significant (*p* < 0.05) and >0.30 [21,30]. In this study, a positive and statistically significant (*p* < 0.0001) correlation was observed between the total scores and total score of variables of the HPSHC and CMFS, which evaluate related conditions (Table 4). It was agreed that there was a “*strong*” association between the total HPSHC and CMFS scores and a “*weak*” and “*moderate*” relationship between the total factor scores. The test–retest analysis of the HPSHC revealed a positive and statistically significant (*p* < 0.0001) correlation between the scale total scores of two assessments and the factor total scores (Table 3). In the repeated measurement, the overall scale scores and the total factor scores did not change despite the passing of two weeks. Therefore, based on the total scores of the two measures and the total scores of the variables, the stability of the scale was “*very strong*”, “*strong*”, and “*moderate*”.

The reliability coefficients of the HPSHC indicate that it possesses the ability to yield consistent outcomes across many instances and when administered by different individuals, provided that the children being assessed share similar sample characteristics. The “HPSHC is a valid and reliable tool that can measure healthy children’s perception of hospitals” hypothesis was validated via the validity and reliability analysis conducted in this study.

**Limitations**

This study had several limitations. The HPSHC was intended to measure the perception of hospitals of children aged 7–10 years. However, 7-year-old healthy children could not be included in the sample because they lack the reading and questionnaire-answering skills necessary. Therefore, the exclusion of children aged 7 constitutes a restriction. On the other hand, it is imperative to remove those who are below the age of seven. Consequently, a representative sample exhibiting shared characteristics was established to ensure the generalizability of the research findings.

In parallel form correlation analyses, the mean ± SD values of the HPSHC and CMFS, which are similar conceptually and in terms of the area of measurement, were equal. However, the equality of variances could not be achieved. In parallel form analysis, the use of scales that cannot provide variance equality is a limitation. In parallel form correlation analyses, the correlation coefficients of two scales with similar conceptual frameworks, measurement domains, numbers of items, means, standard deviation values, and variance values showed that there was a “strong” relationship between the two scales (Table 4). The findings of this study indicate that while a complete equality of variance was not attained, there were no significant changes observed in the measurements of the two scales. This suggests that the measurement technique employed in this study can be considered reliable.

Due to the COVID-19 pandemic, the children’s school attendance reduced, and many returned the scale forms without completing them, making it impossible to recruit enough participants for the second application. Therefore, the construct validity of the scale could not be reconfirmed with confirmatory factor analysis and convergent-discriminant validity analyses. The COVID-19 pandemic led to a decline in the attendance of children at schools in 2021. Notwithstanding the aforementioned circumstances, it is noteworthy that the sample size employed in the study adhered to the established criteria at a modest level (330 individuals ÷ 25 items = 13:1), and thus, a consistent factor structure was established.

**Strengths**

This study had several strengths. One of the research’s strengths is the creation of a measurement tool that can assess perception of hospitals of healthy children, which is lacking in the literature. Furthermore, all phases of the scale development procedure were documented and explored in as much depth as feasible, in the order of their occurrence. This study performed a test–retest analysis, which was cited as a problem in previous studies, and retested the consistency of the scale. According to the results of the validity and reliability investigation, the HPSHC is an instrument that evaluates the perception of hospitals of children accurately and fully.

## 5. Conclusions

The HPSHC was intended to measure the perception of hospitals of children aged 7–10 years. However, 7-year-old healthy children could not be included in the sample because they lacked the required reading and questionnaire-answering skills. In this study, a 25-item, six-factor scale was constructed to assess the perception of hospitals of 8- to 10-year-old healthy children. Through testing, the validity and reliability of the scale’s measurements were confirmed. This is the first scale that can be used to measure the emotional conditions of 8- to 10-year-old healthy and hospitalized children regarding hospital visits or hospitalization. Depending on the academic progress of the child in various societies, the HPSHC can also be administered to seven-year-olds. The utilization of this scale is applicable to healthcare professionals and school nurses operating within various pediatric healthcare settings.

This scale will contribute to the measuring of the perception of hospitals, the implementation of appropriate safeguards, and the planning of treatment and care practices before children develop a negative perception of hospitals and are exposed to their impacts. Furthermore, it is important to establish a comprehensive assessment instrument capable of quantifying the perception of hospitals pertaining to pediatric patients afflicted with chronic illnesses.

## Figures and Tables

**Table 1:** Factor structure of the HPSHC, Cronbach’s alpha, and item–total correlation coefficients (N = 330).

No of Item	Factors, Items and Results	FL	^a^ ITCC
	*Factor 1: Separation, α = 0.76, EV = 24.36%*		
2	When I go to the hospital, I think I will be separated from my friends.	0.76	0.39
1	When I go to the hospital, I think I will be separated from my family.	0.74	0.37
3	When I go to the hospital, I think I will be separated from my best friend.	0.65	0.38
5	When I go to the hospital, I think I will fall behind from my school work and classes.	0.60	0.32
4	When I go to the hospital, I think I will be alone.	0.58	0.46
7	When I go to the hospital, I think I will spend a lot of time there.	0.42	0.57
	*Factor 2: Invasive and non-invasive interventions* *; α = 0.74, EV = 8.16%*		
14	When I go to the hospital, I think I am going to have an injection.	0.75	0.44
15	When I go to the hospital, I think blood will be drawn from my body.	0.72	0.38
13	When I go to the hospital, I think painful or discomforting procedures will be performed to me.	0.57	0.51
16	When I go to the hospital, I think doctors will keep examining me.	0.56	0.46
11	When I go to the hospital, I think tools will be inserted into me.	0.48	0.53
	*Factor 3: Fear and loss of control; α = 0.63, EV = 7.26%*		
9	When I go to the hospital, I think I will be afraid to talk to the nurse and doctor.	0.73	0.36
20	I think the hospital is a scary place.	0.55	0.46
18	When I go to the hospital, I think I will be forced to lie on the examination table.	0.55	0.45
23	I think I will be unhappy in the hospital.	0.49	0.36
17	When I go to the hospital, I think that applications to my body will be made without my permission.	0.46	0.34
	*Factor 4: Body image; α = 0.63, EV = 5.19%*		
12	When I go to the hospital, I think I will have an operation.	0.69	0.46
19	When I go to the hospital, I think I will become disabled.	0.69	0.35
10	When I go to the hospital, I think I will die.	0.63	0.44
	*Factor 5: Physiological needs; α = 0.58, EV = 4.47%*		
26	In the hospital, I think that I will not be able to use the toilet comfortably.	0.79	0.36
25	In the hospital, I think I will be able to play.	0.62	0.42
27	In the hospital, I think I will have to eat foods I do not like.	0.56	0.44
	*Factor 6: Hospital environment; α = 0.58. EV = 4.10%*		
22	I think the hospital is a noisy and crowded place.	0.76	0.36
21	I think of the hospital as a place where words I do not know are used.	0.59	0.39
24	I think there are strangers in the hospital.	0.56	0.40
	***The total scale; α = 0.87, CEV = 53.57%***		

^a^ Pearson product–moment correlation coefficient. Note. FL = factor loading, ITCC = item–total correlation coefficient, α = Cronbach’s alpha coefficient, EV = explained variance, and CEV = cumulative explained variance.

**Table 2:** Internal consistency analyses of the HPSHC (N = 330).

Coefficients	Parts/Forms	Value	^c^ N of the Items
Cronbach’s alpha	Part 1	0.80	^a^ 13
	Part 2	0.77	^b^ 12
Correlation between forms		0.69	
Guttman split-half		0.81	
Spearman–Brown	Equal length	0.82	
	Unequal length	0.82	

^a^ First 13 items of scale, ^b^ last 12 items of scale, and ^c^ total items = 25.

**Table 3:** Test–retest and ICC analyses of the HPSHC (N = 100).

Categories	^a^ Test–Retest		^b^ Intraclass Correlation Coefficient						
				95% Confidence Interval		^b^ F Test			
	^a^ r	*p*	ICC	Lower Bound	Upper Bound	Value	*df*1	*df*2	*p*
Factor 1	0.721	˂0.0001	0.83	0.75	0.89	6.167	99	99	˂0.0001
Factor 2	0.579		0.72	0.59	0.81	3.692			
Factor 3	0.489		0.64	0.47	0.76	2.815			
Factor 4	0.572		0.72	0.53	0.81	3.657			
Factor 5	0.418		0.59	0.39	0.72	2.436			
Factor 6	0.368		0.53	0.31	0.68	2.162			
**Total**	**0.657**		0.79	0.69	0.86	4.942			

^a^ Pearson product–moment correlation coefficient; ^b^ average intraclass measures. ICC = intraclass correlation coefficient; *df* = degree of freedom. Note: a two-way mixed effects model was used.

**Table 4:** Parallel form reliability analysis (N = 330).

HPSHC		CMFS				
	^a^ Test	Factor 1	Factor 2	Factor 3	Factor 4	Total Scale
Factor 1	r	0.195	0.394	0.268	0.323	0.367
Factor 2	r	0.400	0.431	0.426	0.401	0.504
Factor 3	r	0.437	0.528	0.378	0.355	0.519
Factor 4	r	0.268	0.463	0.260	0.269	0.388
Factor 5	r	0.343	0.380	0.398	0.417	0.472
Factor 6	r	0.216	0.259	0.282	0.289	0.321
**Total scale**	r	0.448	0.594	0.487	0.498	0.622
All *p* values are <0.0001 in correlation analysis.						
The 28-item HPSHC: mean ± SD = 46.92 ± 9.88 and variance = 97.733						
The 25-item HPSHC: mean ± SD = 42.81 ± 9.15 and variance = 83.750						
The 29-item CMFS: mean ± SD = 45.24 ± 10.16 and variance = 103.31						

^a^ Pearson product–moment correlation coefficient; SD = standard deviation.

## Data Availability

The datasets generated during and/or analyzed during the current study are available from the corresponding author on reasonable request.

## References

[1] Hockenberry M., Wilson D. (2019). Wong’s Nursing Care of Infants and Children.

[2] Sternberg R.J. (2009). Cognitive Psychology: International Student Edition.

[3] Chappuis M., Vannay-Bouchiche C., Fluckiger M., Monnier M., Cathieni F., Terra R., Piot-Ziegler C. (2011). Children’s experience regarding the quality of their hospital stay: The development of an assessment questionnaire for children. J. Nurs. Care Qual..

[4] Lerwick J.L. (2016). Minimizing pediatric healthcare-induced anxiety and trauma. World J. Clin. Pediatr..

[5] Rokach A. (2016). Psychologial emotional and physical experiences of hospitalized children. Clin. Case Rep. Rev..

[6] Salmela M., Aronen E.T., Salanterä S. (2011). The experience of hospital-related fears of 4- to 6-year-old children. Child Care Health Dev..

[7] Wilson E.M., Megel E.M., Enenbach L., Carlson L.K. (2010). The voices of children stories about hospitalization. J. Pediatr. Health Care.

[8] Li H.C.W., Lopez V., Lee T.L.I. (2007). Effects of preoperative therapeutic play on outcomes of school-age children undergoing day surgery. Res. Nurs. Health.

[9] Li W.H., Chung J.O.K., Ho K.Y., Kwok B.M.C. (2006). Play interventions to reduce anxiety and negative emotions in hospitalized children. BMC Pediatr..

[10] Kalmakis K.A., Chandler G.E. (2014). Adverse childhood experiences: Towards a clear conceptual meaning. J. Adv. Nurs..

[11] Forsner M., Jansson L., Sørlıe V. (2005). The experience of being ill as narrated by hospitalized children aged 7–10 years with short-term illness. J. Child Health Care.

[12] Hedén L., Von Essen L., Ljugman G. (2019). Children’s self-reports of fear and pain levels during needle procedures. Nurs. Open.

[13] Zembar M.J., Blume L.B. (2020). Middle Childhood to Middle Adolescence; a Contextual Approach.

[14] Broome M.E., Hellier A.P. (1987). School-age children’s fears of medical experiences. Issues Compr. Pediatr. Nurs..

[15] Henson R.K., Roberts J.K. (2006). Use of exploratory factor analysis in published research. Common errors and some comment on improved practice. Educ. Psychol. Meas..

[16] Alak V. (1993). Fears and Nursing Practices of Children Aged 7–14 Coming to the Hospital for Surgery. Ph.D. Thesis.

[17] Broome M., Mobley T., Strickland O.L., Dilorio C. (2003). The child medical fears scale. Measurement of Nursing Outcomes Volume 2: Client Outcomes and Quality Care.

[18] Foster R.L., Park J.-H. (2012). An Integrative review of literature examining psychometric properties of ınstruments measuring anxiety or fear in hospitalized children. Pain Manag. Nurs..

[19] San Martín-Rodríguez L., Soto-Ruiz N., Ferraz-Torres M., García-Vivar C., Saralegui-Gainza A., Escalada-Hernández P. (2022). The Spanish version of the child medical fear questionnaire: Cross-cultural adaptation and validation. Int. J. Environ. Res. Public Health.

[20] Almanasreh E., Moles R., Chen T.F. (2019). Evaluation of methods used for estimating content validity. Res. Social Adm. Pharm..

[21] Boateng G.O., Neilands T.B., Frongillo E.A., Melgar-Quiñonez H.R., Young S.L. (2018). Best practices for developing and validating scales for health, social, and behavioral research: A primer. Front. Public Health.

[22] Carpenter S. (2018). Ten steps in scale development and reporting: A guide for researchers. Commun. Methods Meas..

[23] DeVellis R.F., Thorpe C.T. (2022). Scale Development: Theory and Applications.

[24] Ayre C., Scally A.J. (2014). Critical values for Lawshe’s content validity ratio: Revisiting the original methods of calculation. Meas. Eval. Couns. Dev..

[25] Davis L.L. (1992). Instrument review: Getting the most from a panel of experts. Appl. Nurs. Res..

[26] Polit D.F., Beck C.T. (2006). The content validity index: Are you sure you know what’s being reported? Critique and recommendations. Res. Nurs. Health.

[27] Polit D.F., Beck C.T., Owen S.V. (2007). Is the CVI an acceptable indicator of content validity? Appraisal and recommendations. Res. Nurs. Health.

[28] Bandalos D. (2018). L Measurement Theory and Applications for the Social Sciences.

[29] Field A. (2013). Discovering Statistics Using SPSS.

[30] Schober P., Boer C., Schwarte L.A. (2018). Correlation coefficients: Appropriate use and ınterpretation. Anesth. Analg..

[31] Beavers A.S., Lounsbury J.W., Richards J.K., Huck S.W., Skolits G.J., Esquivel S.L. (2013). Practical considerations for using exploratory factor analysis in educational research. Pract. Assess. Res. Eval..

[32] Costello A.B., Osborne J.W. (2005). Best practices in exploratory factor analysis: Four recommendations for getting the most from your analysis. Pract. Assess. Res. Eval..

[33] Osborne J.W., Costello A.B. (2004). Sample size and subject to item ratio in principal components analysis. Pract. Assess. Res. Eval..

[34] Ferrando P.J., Anguiano-Carrasco C. (2010). El análisis factorial como técnica de investigación en psicología [Factor analysis as a technique in psychological research]. Papeles Del Psicól..

[35] Koo T.K., Li M.Y. (2016). A guideline of selecting and reporting intraclass correlation coefficients for reliability research. J. Chiropr. Med..

[36] O’Rourke N., Hatcher L. (2013). A Step-by-Step Approach to Using SAS for Factor Analysis and Structural Equation Modeling.

[37] Kyriazos T.A., Stalikas A. (2018). Applied Psychometrics: The Steps of Scale Development and Standardization Process. Psychology.

[38] Kyriazos T.A. (2018). Applied Psychometrics: Sample Size and Sample Power Considerations in Factor Analysis (EFA, CFA) and SEM in General. Psychology.

[39] Young A., Pearce S. (2013). A beginner’s guide to factor analysis: Focusing on exploratory factor analysis. Tutor. Quant. Methods Psychol..

[40] Clarke S. (2019). Children’s experiences of staying in hospital from the perspectives of children and children’s nurses: A narrative review. Nurs. Health Care.

[41] Delvecchio E., Salcuni S., Lis A., Germani A., Di Riso D. (2019). Hospitalized children: Anxiety, coping strategies, and pretend play. Front. Public Health.

[42] Kleye I., Hedén L., Karlsson K., Sundler A.J., Darcy L. (2021). Children’s individual voices are required for adequate management of fear and pain during hospital care and treatment. Scand. J. Caring Sci..

